# Autonomous Robots for Space: Trajectory Learning and Adaptation Using Imitation

**DOI:** 10.3389/frobt.2021.638849

**Published:** 2021-05-04

**Authors:** R. B. Ashith Shyam, Zhou Hao, Umberto Montanaro, Shilp Dixit, Arunkumar Rathinam, Yang Gao, Gerhard Neumann, Saber Fallah

**Affiliations:** ^1^ Department of Electrical and Electronic Engineering, Surrey Space Center, University of Surrey, Guildford, United Kingdom; ^2^ Department of Mechanical Engineering, University of Surrey, Guildford, United Kingdom; ^3^ Karlsruhe Institute of Technology, Karlsruhe, Germany

**Keywords:** motion planning, probabilistic movement primitives, robot manipulation, learning from demonstrations, trajectory adaptation

## Abstract

This paper adds on to the on-going efforts to provide more autonomy to space robots and introduces the concept of programming by demonstration or imitation learning for trajectory planning of manipulators on free-floating spacecraft. A redundant 7-DoF robotic arm is mounted on small spacecraft dedicated for debris removal, on-orbit servicing and assembly, autonomous and rendezvous docking. The motion of robot (or manipulator) arm induces reaction forces on the spacecraft and hence its attitude changes prompting the Attitude Determination and Control System (ADCS) to take large corrective action. The method introduced here is capable of finding the trajectory that minimizes the attitudinal changes thereby reducing the load on ADCS. One of the critical elements in spacecraft trajectory planning and control is the power consumption. The approach introduced in this work carry out trajectory learning offline by collecting data from demonstrations and encoding it as a probabilistic distribution of trajectories. The learned trajectory distribution can be used for planning in previously unseen situations by conditioning the probabilistic distribution. Hence almost no power is required for computations after deployment. Sampling from a conditioned distribution provides several possible trajectories from the same start to goal state. To determine the trajectory that minimizes attitudinal changes, a cost term is defined and the trajectory which minimizes this cost is considered the optimal one.

## 1 Introduction

Robots that operate in space are very much limited due to the unique challenges encountered like communication latency, lack of power sources and extreme safety requirements. Current robots operating in space are either controlled from ground stations or tele-operated. As a result, there is large scale research going on to make space robots more autonomous.

One of the critical issues that require immediate attention is the ever increasing space junk, especially in the past decade as it poses a huge threat to the functioning spacecraft (satellites, International Space Station etc.). There are more than half a million debris in Low Earth Orbit (LEO) NASA, 2013 and it is estimated that the space environment can be stabilised when on the order of 5–10 objects are removed from LEO per year ESA, 2018. Although several methods for space debris removal has been proposed like harpoons, nets, tentacles SPACE.COM, 2018, using robotic arms to capture still remain the preferred choice as it can be extended to various other application areas like on-orbit servicing and assembly and autonomous rendezvous and docking.

Spacecrafts require flying at a nominal attitude to charge battery, communicate with the ground station and determine its attitude and position. However orbital environment being micro-gravitational poses a difficult challenge since the spacecraft bus to which the robot-arm is attached is floating and any motion of the robot-arm would induce an attitudinal disturbance to the spacecraft. For free-flying spacecraft, the attitude determination and control system (ADCS) continuously compensate for the disturbances from the operation of the manipulator to maintain the nominal attitude of the spacecraft and hence a lot of energy is consumed.

Free-floating is a conceptual operating state of the spacecraft (when ADCS is switched off) installed with robotic arm. This type of spacecraft leave the attitude uncontrolled during the operation of the robotic arm. However, leaving the attitude of the spacecraft tumbled is unsafe and not ideal for the power system and the sensors used for determining its attitude. For both cases, we want the trajectory planner of the robotic arm to consider two important aspects, viz.,

1. minimal attitudinal disturbances of the spacecraft bus due to manipulator operation
2. computationally inexpensive for minimal power consumption.

Inthis work, we demonstrate how programming by demonstrations or imitation learning Shyam et al., 2019; Paraschos et al., 2018 can be used to plan trajectory of a 7-DoF robot arm attached to small spacecrafts. The learned trajectories are efficiently encoded as a probabilistic distribution (PD) from which we can sample out trajectories for reproduction. This method is computationally efficient and is capable of minimizing the attitude disturbances (as shown in Section 5.3).

Optimal control methods Rybus et al., 2016; Rybus et al., 2017; Camacho and Alba, 2013 are well developed but it often get stuck at local minima due to poor initial guess and if successful, produces only a single trajectory. However, modeling as a PD captures the mean as well as the variance of the trajectories. The variance information could be used for sampling initial guess values form the PD for optimization based local planners to perturb the trajectory to avoid obstacles. It is known that the quality of initial guess determines the computational load and avoidance of local minima Shyam et al., 2019.

Future space robots is expected to have human arm like dexterity. Hence it is proposed to use a 7-DoF redundant robot arm. This has the added advantage that the planning and control can still be carried out effectively even if a joint encoder or sensor fails.

## 2 Related Work

The analysis of the kinematic and dynamic of spacecraft with manipulator is well established. Exploiting the non-holonomic behavior of the orbital manipulator system for spacecraft attitude and end-effector trajectory control have been studied extensively. It usually involves joint space techniques to control both the motion of the arm and sometimes the spacecraft attitude Yoshida and Nakanishi, 2003; Hirano et al., 2018. Early research used mapping methods to correlate the end-effector position with the induced disturbances on the spacecraft to minimize the attitude disturbances Torres and Dubowsky, 1992; Vafa and Dubowsky, 1993. However, the mapping methods are computationally inefficient and furthermore, higher DoF manipulators will significantly increase the mapping difficulty and are challenging to find optimised paths.

The work by Nenchev et al. Nenchev et al. (1999) proves that for certain manipulator motions, no reaction forces are induced on the spacecraft. As mentioned in their work, such solutions exists only for some special cases where integrability of the reaction null space velocity exists. This work then inspired many following research to exploit and optimise the control method for spacecraft with manipulator Dimitrov and Yoshida, 2006; Piersigilli et al., 2010; Nguyen-Huynh and Sharf, 2013.

More recently, researchers have attempted to solve the problem of trajectory planning by minimizing a cost functional which satisfies certain criteria. For example Rybus et al. (2016); Seweryn and Banaszkiewicz (2008) minimizes the power consumption. Non-linear Model Predictive Control (NMPC) have been used for control of free-floating spacecrafts Rybus et al., 2017 but it remains to be seen how such heavy computations can be carried out by an on-board spacecraft computer. The other focus is post-panning impedance control of the orbital manipulator to free-motion targets. These researches aim to solve the kinodynamics in order to finely control the impact force for safe and accurate manipulation in the micro-gravity environment Papadopoulos, 1992.

### 2.1 Contributions

The main contributions of this work are

1. Imitation learning based trajectory planning:

• First the trajectories are learned from demonstrations and encoded as a probabilistic distribution (PD). Planning to an unseen target only requires sampling and conditioning of the PD. This avoids computationally expensive optimization methods (which usually have a cost function to minimize) to run on on-board computer.

2. Minimize attitude disturbances during capture:

• Sampling from a PD for our redundant manipulator arm can produce infinite possible trajectories theoretically. Attitude disturbances for each trajectory can be easily computed and is possible to choose the trajectory with the least disturbance.

This paper is organized as follows. Section 3 gives briefly kinematic and dynamic formulations of the spacecraft manipulator system. In Section 4, we discuss the method used for generating trajectory data for learning. Section 5 provides the equations by which trajectories can be compactly encoded as a probabilistic distribution which can be used further for reproduction to unseen situations. Section 6 presents the simulation results and Section 7 gives the conclusions and future directions.

## 3 Dynamic Formulation

The kinematic and dynamic formulation of free-floating spacecrafts have been studied previously Umetani and Yoshida, 1989; Wilde et al., 2018; Nanos and Papadopoulos, 2017. Here we give a abridged version of the same for completeness. This formulation makes it easier to compute various matrices especially the coriolis and centrifugal which requires symbolic differentiation of the mass matrix. The whole formulation is carried out using Python’s symbolic library called ‘sympy’ Meurer et al., 2017 which supports ‘C’ code generation as well for faster execution.

### 3.1 Nomenclature

•  $m_{i}$: mass of the *i* ^*th*^ link, the first being the spacecraft
•  $r_{i}$: position vector of the centre of mass of *i* ^*th*^ link with respect to the inertial co-ordinate system
•  ${\dot{r}}_{i}$: linear velocity of the centre of mass of *i* ^*th*^ link with respect to the inertial co-ordinate system
•  $I_{i}$: moment of inertia of *i* ^*th*^ link with respect to the inertial co-ordinate system
•  $\omega_{i}$: angular velocity of the *i* ^*th*^ link with respect to the inertial co-ordinate system
•  $a_{i}$: vector pointing from the joint *i* to the centre of mass of link *i*
•  $b_{i}$: vector pointing from the centre of mass of link *i* to joint *i* + 1
•  $l_{i}$: length of *i* ^*th*^ link
•  $\phi_{s}$: vector of attitude angles (yaw, pitch and roll) of the spacecraft
•  $\phi_{m}$: vector of manipulator joint angles

### 3.2 Assumptions

1. Momenta is conserved and is zero at the beginning
2. Gravity is negligible
3. The Centre of Mass of the system coincides with the origin of the inertial co-ordinate system
4. The motion planning is carried out when the satellite-manipulator system at a safe state and is sufficiently close to the target.

The mass centre of the spacecraft-manipulator arm can be described as

$$\sum_{i=0}^{n}m_{i}r_{i}=0$$ (1)

The linear and angular momentum conservation equations become

$$\sum_{i=0}^{n}m_{i}{\dot{r}}_{i}=0$$ (2)

$$\sum_{i=0}^{n}I_{i}\omega_{i}=0$$ (3)

**FIGURE 1:**
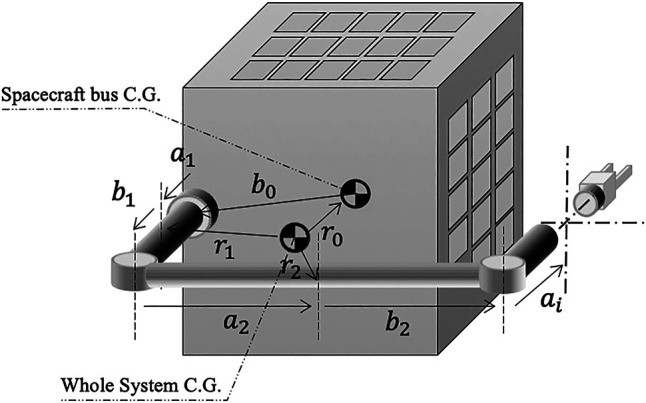
Schematic diagram of a spacecraft-manipulator arm.

From Figure 1, the geometrical relationship between the various vectors can be written as

$$r_{i}=r_{i-1}+a_{i}+b_{i-1}$$ (4)

Equations. 1, 4 can be solved simultaneously to obtain the centre of mass of the spacecraft and can be expressed as in Eq. 5

$$\begin{array}{l} r_{s}=r_{0}=-\sum_{i=0}^{n-1}Kij\left(b_{i}+a_{i+1}\right)\\ K_{ij}=1-\sum_{j=0}^{i}\frac{m_{j}}{W} \end{array}$$ (5)

$$v_{s}=\frac{d}{dt}r_{s}$$ (6)

where *W* is the total mass of the system, $r_{s}$ and $v_{s}$ are the position vector and linear velocity of the spacecraft with all vectors expressed with respect to the inertial co-ordinate system. The position vector and velocity of the rest of the links can be found using the recursive relation given by Eq. 4. The differential kinematics of the satellite-manipulator arm system gives the jacobian matrix of the system which consists of the manipulator part (${\text{J}}_{m}$) and the satellite part ($J_{s}$). Thus the end-effector velocity, $v_{eef}$, and the momentum conservation can be expressed as in Umetani and Yoshida, 1989.

$$v_{eef}=J_{s\dot{\phi }s}+J_{m\dot{\phi }m}$$ (7a)

$$0=I_{s}{\dot{\phi }}_{s}+I_{m}{\dot{\phi }}_{m}$$ (7b)

From Eq. 7, the end-effector velocity can be solved as a function of the manipulator joint rates and generalized jacobian, *J* ^*^ given by $\left(J_{m}-J_{s}I_{s}^{-1}I_{m}\right)$

$$\begin{array}{c} v_{eef}=\left(J_{m}-J_{s}I_{s}^{-1}I_{m}\right){\dot{\phi }}_{m}\\ \;\;=J^{*}{\dot{\phi }}_{m} \end{array}$$ (8)

where $I_{s}$ and $I_{m}$ are respectively the satellite and manipulator inertia matrices expressed in inertial co-ordinate system Umetani and Yoshida, 1989.

The Kinetic energy, T, can then be expressed as

$$T=\sum_{i=0}^{n}mi\left(vi\cdot vi\right)=\frac{1}{2}{\dot{\phi }}^{T}M\left(\phi \right)\dot{\phi }$$ (9)

where M(ϕ) is the mass matrix and $\phi ={\left[\phi_{s}^{T}\phi_{m}^{T}\right]}^{T}$. The centripetal and coriolis vector, C, is given by

$$\;C\left(\phi ,\dot{\phi }\right)=\dot{M}\left(\phi \right)\;\dot{\phi }-\frac{1}{2}\left[\begin{array}{c} {\dot{\phi }}^{T}\frac{\partial M\left(\phi \right)}{\partial \phi_{1}}\dot{\phi }\\ {\dot{\phi }}^{T}\frac{\partial M\left(\phi \right)}{\partial \phi_{2}}\dot{\phi }\\ .\\ .\\ {\dot{\phi }}^{T}\frac{\partial M\left(\phi \right)}{\partial \phi_{n}}\dot{\phi } \end{array}\right]\;$$ (10)

The equation of motion of the free-floating spacecraft manipulator system can be written as

$$M\left(\phi \right)\ddot{\phi }+C\left(\phi ,\dot{\phi }\right)=\left[\begin{array}{c} 0\\ \tau \end{array}\right]$$ (11)

where τ is the control torque to be applied at the manipulator joints.

## 4 Data Generation for Trajectory Learning

**FIGURE 2:**
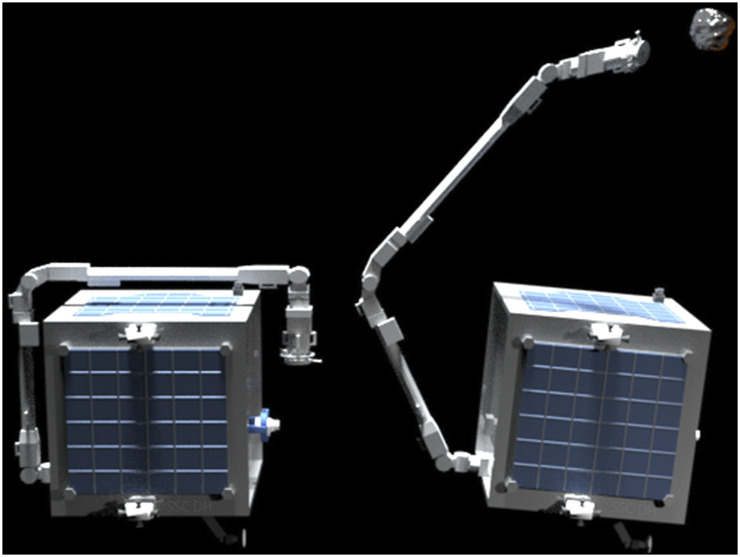
**Left:** Home position of the Future Space Debris Removal Orbital Manipulator (FSDROM) (a 7-DoF redundant robot arm attached to the spacecraft); **Right:** Position of the Future Space Debris Removal Orbital Manipulator (FSDROM) with Manipulator capturing an orbital debris.

The method introduced here requires data samples for trajectory learning. A trajectory, ζ, is a mapping of all the robot configuration (*x*) from start to goal with time. Mathematically it can be represented as ζ:[0,1]→x where $x\in {\mathbb{R}}^{d}$ and *d* corresponds to the number of joints with ζ(0) and ζ(1) being the start and goal configurations respectively. These trajectories could be generated by a human expert by demonstrations Zhu and Hu, 2018; Havoutis and Calinon, 2019. As real hardware orbital simulation of micro-gravity environment being extremely expensive, we demonstrate the concept by generating trajectories using an optimal control algorithm Kirk, 2004. We make use of the redundancy of the chosen 7-DoF manipulator arm to generate several trajectories which starts at the home position (Figure 2) and go to a particular goal state given by the vision system. Once enough trajectories are generated, the goal state is changed and the process is repeated until the entire workspace is covered.

The cost function for trajectory generation is given as

$$J=x_{T}^{T}P_{t}x_{T}+\int_{t_{0}}^{T-1}x_{t}^{T}Q_{t}x_{t}+u_{t}^{T}R_{t}u_{t}\;dt$$ (12)

subject to the constraints

$$\begin{array}{l} {\dot{x}}_{t}=A_{t}x_{t}+B_{t}u_{t}\\ x_{t}\left(0\right)=x_{0} \end{array}$$

where $x_{t}$ represents the state (position and velocity in task space) of the manipulator joints at time *t*. For a space-manipulator, once the manipulator states have been found out, the satellite states can be determined from eq. 7 and integration. Here $Q_{t}$ and $R_{t}$ are respectively the time varying state and control cost matrices, $P_{t}$ is the stabilizing matrix obtained by the solution of algebraic Ricatti equation at every time instant, $t_{0},T$ are the initial and final time respectively. The trajectory data samples in simulation are obtained by the following methods.

1. varying the cost matrices thus encouraging certain joint motions and discouraging certain other joint motions.
2. introducing artificial obstacles (elastic bands Quinlan and Khatib (1993)) between the start and goal point so as to force the redundant robot to follow a different trajectory to the same goal point
3. introduction of noise into the system given by Eq. 12.

## 5 Trajectory Encoding and Reproduction

The generated trajectory data samples need to be represented in an efficient manner for future planning and control. It has to be mentioned that the trajectory planning is carried out when the spacecraft-robot arm is sufficiently close to the target and is safe to operate. Here we demonstrate the core idea of this work by representing the generated trajectories as a probabilistic distribution. We find that Gaussian distributions fit all the essential criteria for efficiently representing trajectories as it depends only on two parameters i.e. mean and covariance. For reproduction of trajectories to unseen situations, we use the conditioning property of the Gaussian as explained in Section 5.2.

### 5.1 Gaussian Trajectory Encoding

The encoding of the trajectories can be expressed as a linear basis function model as in Eq. 13 where$\psi_{t}$ = ${\left[\psi_{t}\;\dot{\psi_{t}}\right]}^{T}$ is the basis function (see APPENDIX A), *w* a parameter vector, plus some error ε. Such a representation reduces the number of parameters and facilitates learning. Assuming trajectories to be independent and identically distributed, the probability of observing a trajectory, ζ, given the parameter vector *w* can be written as in Eq. 14 Paraschos et al., 2018.

$$x_{t}=\left[\begin{array}{c} q_{t}\\ {\dot{q}}_{t} \end{array}\right]=\left[\begin{array}{c} \psi_{t}\\ {\dot{\psi }}_{t} \end{array}\right]w+\varepsilon_{x}$$ (13)

$$p\left(\zeta |w\right)=\underset{t}{\prod }N\left(x_{t}|\psi_{t}w,\Sigma_{x}\right)$$ (14)

where $\epsilon_{x}∼N\left(0,{\text{Σ}}_{x}\right)$ represents the zero-mean Gaussian noise associated with each observation and $x_{t}$ is the state. There are several possible choices for the basis function. Here a radial basis function (or squared exponential) is used for representing stroke based movements which is ideal for motion planning Paraschos et al. (2018). The parameter vector *w* is modeled as another Gaussian distribution with parameter $\theta \;=\left\{\mu_{w},\Sigma_{w}\right\}$ to capture the variance of the trajectories. (Here $\mu_{w}$ and ${\text{Σ}}_{w}$ are respectively the mean and covariance of the Gaussian). Using the linear transformation property of the Gaussian distribution (see APPENDIX B), the state can be represented asFor the generated trajectory samples, the parameter vector *w* can be estimated as a ridge regression and is given in Eq. 16

$$\begin{array}{c} p\left(x_{t};\theta \right)=\int N\left(x_{t}|\psi_{t}w,\Sigma_{x}\right)N\left(w|\mu_{w},\Sigma_{w}\right)dw\\ =N\left(x_{t}|\mu_{w},\psi_{t}\Sigma_{w}\psi_{t}^{T}+\Sigma_{x}\right) \end{array}$$ (15)

$$w_{i}={\left(\Psi^{T}\Psi +\lambda I\right)}^{-1}\;\Psi^{T}X_{i}$$ (16)

where $X_{i}$ is a 1-D concatenated vector (see APPENDIX C) of all joint values during all time steps from the $i^{th}$ trajectory sample and Ψ is a block diagonal matrix with each block diagonal being $\psi_{t}$. The mean and variance of the parameter vector, *w*, are estimated as in Eq. 17

$$\begin{array}{l} \mu_{w}=\frac{1}{N}\overset{N}{\underset{i=1}{\sum }}w_{i}\\ \Sigma_{w}=\frac{1}{N}\overset{N}{\underset{i=1}{\sum }}\left(w_{i}-\mu_{w}\right){\left(w_{i}-\mu_{w}\right)}^{T} \end{array}$$ (17)

where *N* is the number of demonstrations.

All the above computations could be carried out once the spacecraft manipulator design is complete and the whole trajectory planning problem could then be stated as taking the robot from ζ(0) (home position) to ζ(1) which is the pose of the target estimated by the vision system.

### 5.2 Trajectory Planning to Unseen Situations

The data generation described in Section 4 needs several trajectory samples to accurately represent the workspace of the spacecraft-manipulator. However, workspace can have infinite possible locations of the target theoretically and it is impossible to do data generation for all possible goal poses. The Gaussian distribution introduced above can solve this problem by using the conditional distribution property. A probabilistic trajectory distribution can be conditioned to follow not only the desired start and goal state but also the via-points Paraschos et al., 2018. For example, if our trajectory has to pass through a desired state *x* ^*^_t_ the new mean and variance of the conditioned trajectory will be

$$\begin{array}{l} \mu_{w}^{\left[new\right]}=\mu_{w}+L\left(x_{t}^{*}-\psi_{t}^{T}\mu_{w}\right)\\ \Sigma_{w}^{\left[new\right]}=\Sigma_{w}-L\psi_{t}^{T}\Sigma_{w} \end{array}$$ (18)

where *L* is

$$L=\Sigma_{w}\psi_{t}{\left(\Sigma_{x}^{*}+\psi_{t}^{T}\Sigma_{w}\psi_{t}\right)}^{-1}$$ (19)

and ${\text{Σ}}_{x}^{\text{*}}$ is the desired accuracy to which the state ($x_{t}^{\text{*}}$) is to be reached.

### 5.3 Cost of a Trajectory

The cost of a trajectory is a scalar which estimates how much the attitude of the spacecraft changes when the robot arm follows a particular trajectory. It is defined as follows.

$$Q=c^{2}\;\Sigma \;{\dot{\phi }}_{s}^{T}\;{\dot{\phi }}_{s}+\Sigma \;v_{s}^{T}v_{s}$$ (20)

where ${\dot{\phi }}_{s}$ (from Eq. 7b) and $v_{s}$ (from Eq. 6) are respectively the rate of change of Euler angles^1^ and linear velocities of the spacecraft calculated at each discrete time step from initial to final pose, *c* is a angular to linear conversion coefficient which allows to combine an angular value with a linear value and Σ is the summation symbol. The minimum cost corresponds to the trajectory having minimal disturbances.

### 5.4 Algorithm

The algorithm can be summarised as given below.

Algorithm 1: Algorithm for finding optimal trajectory using imitation learning

**Table:** 

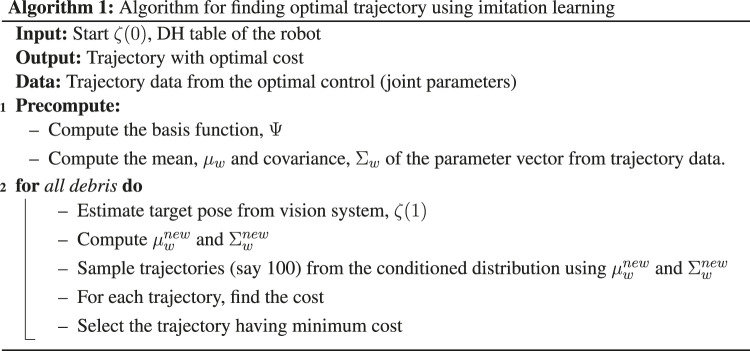

## 6 Simulation Results

The Denavit-Hartenberg Hartenberg and Denavit (1955) parameters of the robot arm is shown in Table 1 and the parameters used for the simulation are shown in Table 2. For this particular result presented here, the end-effector of the robot is commanded to a target position of [−2, 0, 0] m in Cartesian frame.

**TABLE 1:** DH parameters of the robot arm.

Joint	α (rad)	*a* (m)	*d* (m)	θ (rad)
1	$-\frac{\pi }{2}$	0.0	0.5	$\theta_{1}$
2	$\frac{\pi }{2}$	0.0	0.0	$\theta_{2}$
3	$\frac{\pi }{2}$	0.9	0.0	$\theta_{3}$
4	$-\frac{\pi }{2}$	0.9	0.0	$\theta_{4}$
5	$\frac{\pi }{2}$	0.8	0.0	$\theta_{5}$
6	$-\frac{\pi }{2}$	0.8	0.0	$\theta_{6}$
7	$\frac{\pi }{2}$	0.0	0.8	$\theta_{7}$

**TABLE 2:** Simulation Parameters.

	Satellite	L 1	L 2	L 3	L 4	L 5	L 6	L 7
	L 0							
Mass (kg)	200.0	20.0	30.0	30.0	20.0	20.0	20.0	20.0
$I_{x}$	1400.0	0.10	0.25	0.25	0.25	0.25	0.25	0.25
$I_{y}$	1400.0	0.10	25.0	25.0	25.0	25.0	25.0	25.0
$I_{z}$	2040.0	0.10	25.0	25.0	25.0	25.0	25.0	25.0

**FIGURE 3:**
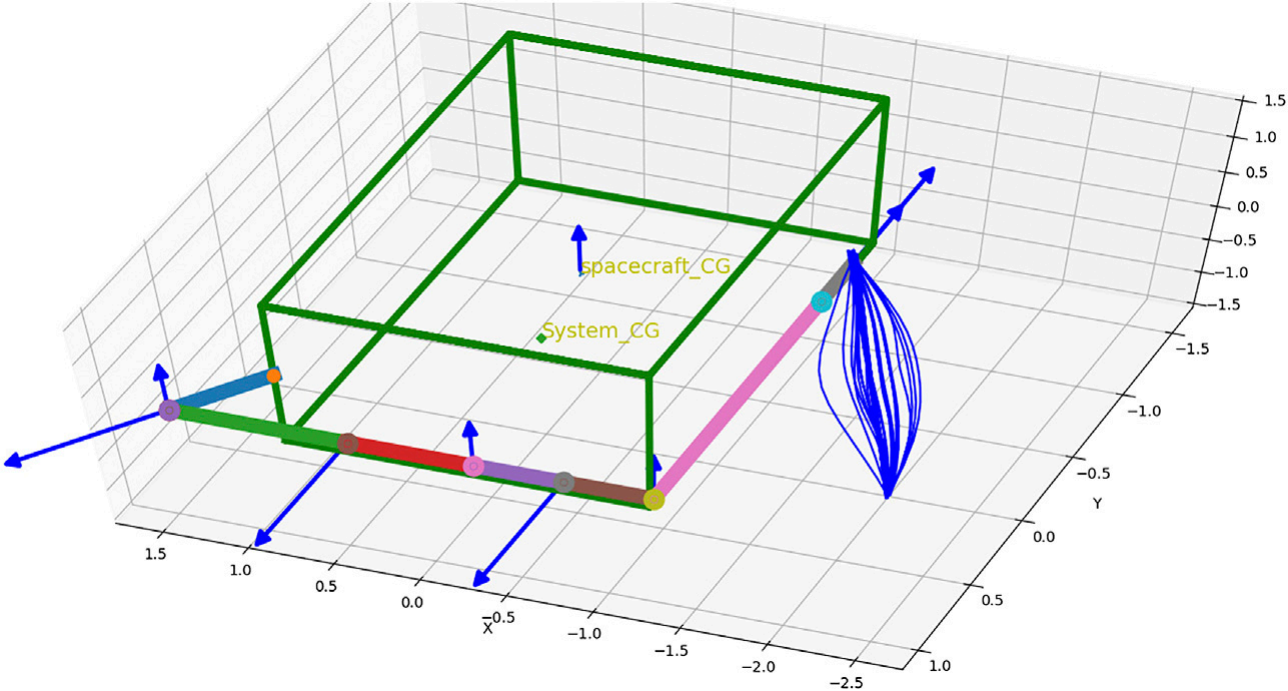
Home position and several possible trajectories of the end-effector to reach the same target.

Figure 3 shows the home position of the robot arm attached to the spacecraft corresponding to the joint values, $\left(0.0,5\frac{\pi }{4},0.0,0.0,\frac{\pi }{2},-\frac{\pi }{2},0.0\right)$. The spacecraft’s Euler angles are (0, 0, 0) at the start and is commanded to any Cartesian position in the world which is considered safe to carry out the manipulation. Equation 6 can be used to find the distance of centre of mass from inertial coordinate system.

**FIGURE 4:**
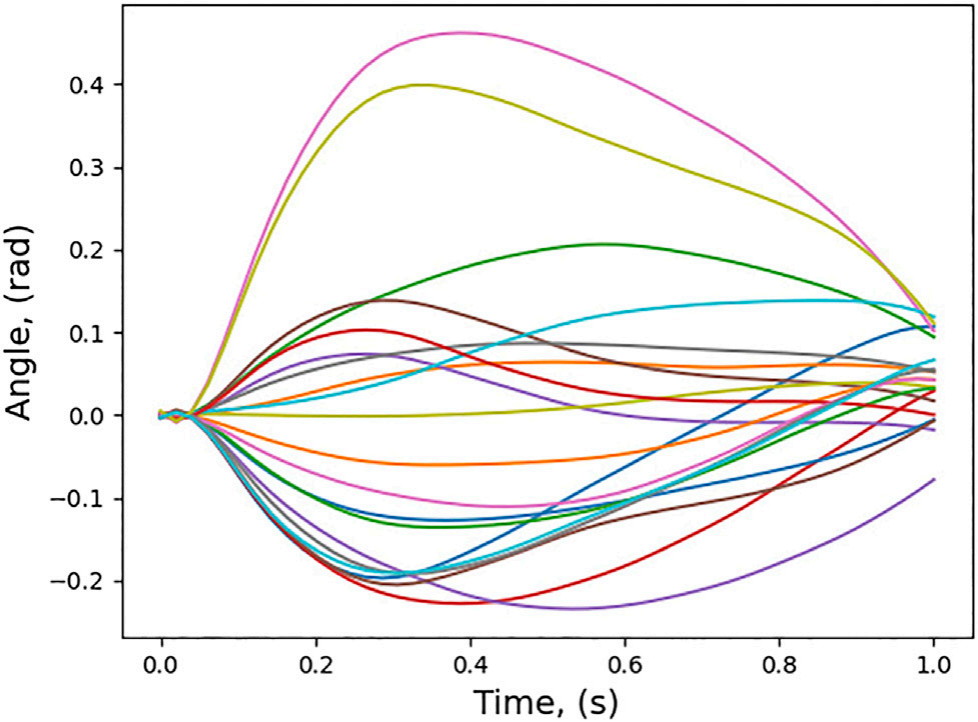
Learned and normalized trajectory distribution for joint one.

The trajectories obtained from the optimal control algorithm are normalized in the time interval 0–1. The learned trajectory distribution for joint 1 is shown in Figure 4.

**FIGURE 5:**
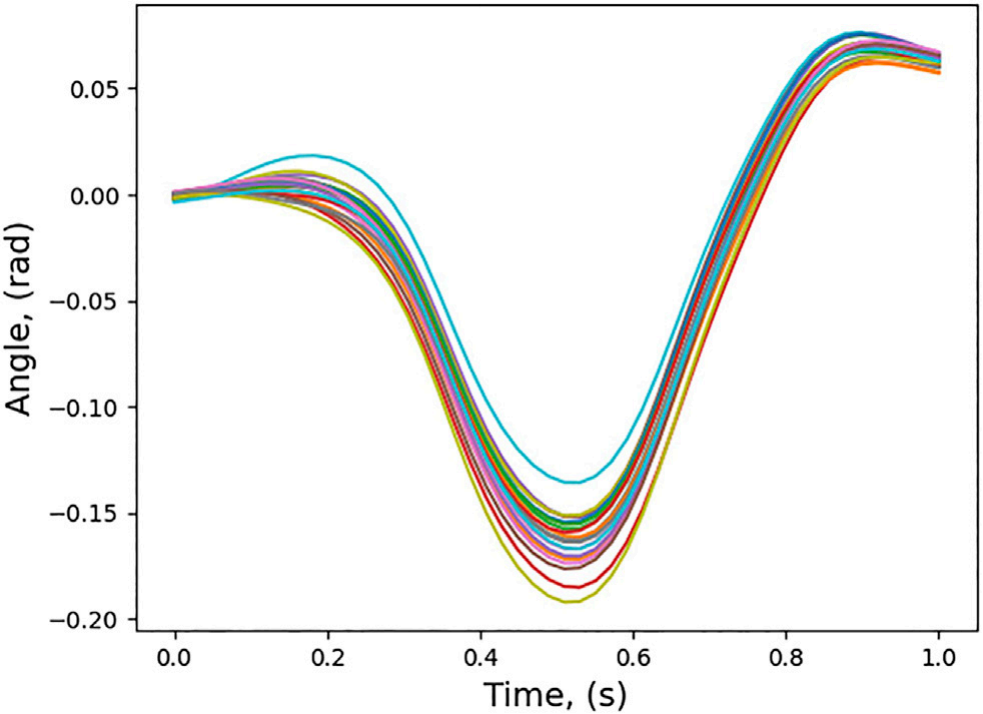
Conditioned trajectory distribution for joint one.

The conditioned trajectory for joint 1 is also shown in Figure 5.

**FIGURE 6:**
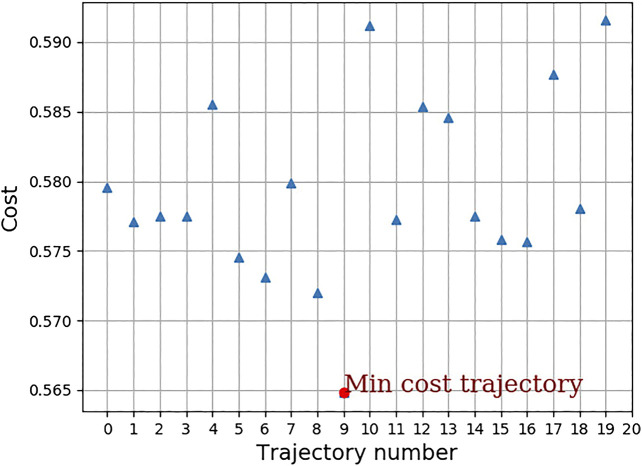
Cost associated with each of the sampled trajectory.

**FIGURE 7:**
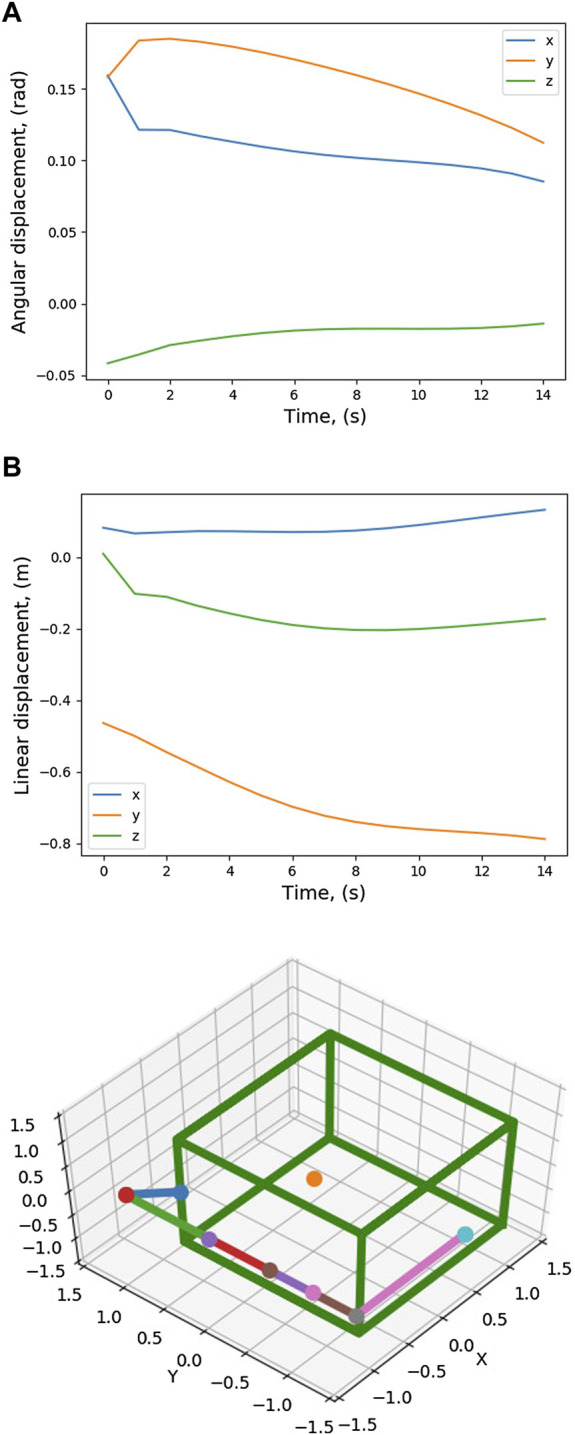
Pose variation of the spacecraft CG during trajectory tracking.

Twenty trajectories in joint space are sampled out from the conditioned distribution. For the twenty joint space trajectories, the corresponding end-effector trajectories in task space are found out and are also shown in Figure 3. Figure 6 shows the cost of each of the trajectories and the trajectory which has the minimum cost. For this trajectory, the induced motion on the spacecraft is shown in Figure 7.

## 7 Conclusion

To the authors’ knowledge, this is the first time imitation learning is used in trajectory planning of robot arms for free floating spacecraft. This work addresses the issue of minimizing attitude disturbance spacecraft bus when the arm reaches out to capture a debris. The learning is carried out offline and is computationally very efficient for finding new trajectories after deployment.

The trajectory learning algorithm presented in this paper will be potentially tested on a Future Space Debris Removal Orbital Manipulator which has a similar micro-satellite spacecraft bus as RemoveDEBRIS Forshaw et al., 2017 but with a 7-DoF redundant robot arm attached, as shown in Figure 2. This is the next step towards space autonomy for on-orbit operations that will be demonstrated by a potential mission concept that goes beyond RemoveDEBRIS spacecraft. The overall mission objectives would be to execute pose estimation, trajectory and motion planning of the robotic arm, and capture a sample debris in order.

## Figures and Tables

**FIGURE A1:**
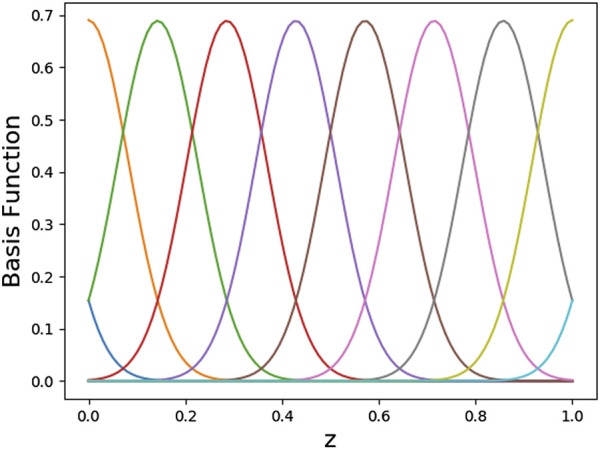
Radial basis function.

## Data Availability

The work undertaken comes under the purview of University of Surrey’s policy. The codes used for generating data can only be made available with prior permission from the university. Please contact Dr. Ashith Shyam Babu at shyamashi@gmail.com for any queries.

## Footnotes

1 It is to be noted that angular velocity and rate of change of Euler angles are not the same. Interested readers may please refer to chapter 3 in Siciliano et al., 2010 for a detailed discussion

## Appendix

### A Basis Function

The basis function used in this work is given by

$$\psi_{i}\left(z\right)=\mathrm{ex}p^{\left(-\frac{{\left(z-c_{i}\right)}^{2}}{h^{2}}\right)}$$

here $z_{t}$ is the phase, $c_{i}$ is the center and *h* is the bandwidth factor. Interested readers can refer to Paraschos et al. (2018) for details. Figure A1 gives a plot of ten basis function centered at [−0.142, 0., 0.142, 0.285, 0.428, 0.571, 0.714, 0.857, 1., 1.142].

### B Linear Transformation Property of Gaussian Distribution

If a random variable *x* is normally distributed (N(μ,Σ)), the linear transformation Ax+c follows the distribution

$$Ax+c∼N\left(A\mu +c,A\Sigma A^{T}\right)$$

### C Explanation of the Learning Scheme

To illustrate the learning process, a simple example of 2-DoF planar robot is considered here. Let $\theta_{1}$ and $\theta_{2}$ represent the joint angles. The complete trajectory for a single demonstration, ζ, concatenated as a 1-D vector is represented as

$$\begin{array}{l} \lambda_{1}=\left[\theta_{1_{t1}},\theta_{1_{t2}},\ldots ,\theta_{1_{tn}}\right]\\ \lambda_{2}=\left[\theta_{2_{t1}},\theta_{2_{t2}},\ldots ,\theta_{2_{tn}}\right] \end{array}$$

*ζ* $={\left[\lambda_{1}\lambda_{2},{\dot{\lambda }}_{1},{\dot{\lambda }}_{2}\right]}_{tn\times 2nDoF}^{T}$

Here $t_{i}$ are the time points, tn and nDoF are the total time points and number of degrees of freedom respectively. Let the number of basis functions be nBf. The matrices $\psi_{t}$ and ${\dot{\psi }}_{t}$ are of dimension 2nDoF×(nBf×2nDoF) each and the matrix Ψ is of dimension (tn×2nDoF)×(nBf×2nDoF). The learning parameter, $w_{i}$ for one demonstration is then calculated by formulating as a regression problem and reducing the loss, ${\left(\zeta -\text{Ψ}w\right)}^{2}$
